# MIS-TLIF or CLIF for single segmental lumbar degenerative disease

**DOI:** 10.1097/MD.0000000000031534

**Published:** 2022-11-04

**Authors:** Shaxika Nazierhan, Chenxi Li, Rui Guo, Linsong Lu, Dilimulati Aikeremu, Kuo Xu, Hao Wang

**Affiliations:** a Department of Orthopedics, People’s Hospital of Xinjiang Uygur Autonomous Region, Urumqi, Xinjiang Uygur Autonomous Region, China; b Oncological Department of Oral and Maxillofacial Surgery, the First Affiliated Hospital of Xinjiang Medical University, School/Hospital of Stomatology Xinjiang Medical University, Stomatological Research Institute of Xinjiang Uygur Autonomous Region, Urumqi, Xinjiang Uygur Autonomous Region, China.

**Keywords:** degenerative, minimally invasive surgery, spondylolisthesis, transforaminal lumbar interbody fusion

## Abstract

We aimed to compare the effect of minimally invasive transforaminal lumbar interbody fusion (MIS-TLIF) and Crenel lateral interbody fusion (CLIF) on single segmental lumbar degenerative disease. Patients with single segmental lumbar degenerative disease undergoing MIS-TLIF (n = 28) and CLIF (n = 28) were enrolled from April to October 2017. Preoperative medical history, anthropometric data, and clinical data were recorded. Visual analogue scores and Oswestry disability index (ODI) were assessed. Radiography was performed before and after surgery. X-ray films were evaluated according to the Bridwell method, visual analogue scores and ODI scores were evaluated. There were no significant differences in the gender, age, clinical diagnosis, involved segment or preoperative ODI score between 2 groups (*P* > .05). During 12-month follow-up, MIS-TLIF group had less intraoperative blood loss, drainage, postoperative bedridden time, and hospital stay (*P* < .05), but more operation time and radiation exposure time compared with CLIF group (*P* < .05). CLIF group reported less pain than MIS-TLIF group (*P* > .05). Both groups had similar lumbar fusion rate (*P* > .05). Overall, CLIF has less complications, less trauma and faster recovery for the treatment of single segmental lumbar degenerate disease when compared with MIS-TLIF. Evaluation of more patients and long-term follow-up are still needed to further validate our findings.

## 1. Introduction

Lumbar degenerative disease is a chronic, degenerative spinal disease, most of the age group is distributed over 50 years old, mainly causing the back pain and symptoms of lower extremity radiation pain, seriously affecting the quality of life. Undoubtedly the most problematic topics of the spinal surgery is to get single segmental lumbar fusion. Transforaminal lumbar interbody fusion (TLIF) is 1 of the important treatments. Foley et al reported that minimally invasive transforaminal lumbar interbody fusion (MIS-TLIF) has achieved excellent therapeutic effects in European and American populations.^[1,2]^ With the emergence of many surgical methods, a new way to treat lumbar degenerative diseases--Crenel Lateral Interbody Fusion (CLIF), also known as cat’s eye lateral lumbar fusion, it is invented by the Second Affiliated Hospital of Zhejiang University. Although both of the 2 minimally invasive surgical procedures can effectively reduce tissue trauma, no comparative analysis has been made so far regarding their clinical outcomes. To the best of our knowledge, this article is the first study to demonstrate the minimum 1-year follow-up of clinical and radiological results following MIS-TLIF surgery and CLIF surgery in treating of single-segment lumbar degenerative diseases.

## 2. Materials and Methods

A total of 56 cases were selected from April 2017 to October 2017. With single-segment lumbar degenerative disease, a single surgeon performed MIS-TLIF surgery and CLIF surgery. In MIS-TLIF group, including 12 males and 16 females; the average age of the patient 50.6 ± 4.8 years (age range – years); 6 cases of lumbar degenerative disease (5 cases of grade I, 1 case of degree II), 7 cases of lumbar spinal stenosis, lumbar disc herniation with lumbar instability 15 cases. In the same period, 28 patients with single-segment lumbar degenerative disease treated with CLIF were included in the control group (CLIF group), including 14 males and 14 females; mean age 51.2 ± 3.3 years (age range – years); 7 cases of lumbar spondylolisthesis (I degree 7 cases), 13 cases of lumbar spinal stenosis, lumbar disc herniation with lumbar instability in 8 cases.

Antibiotics were routinely used for 48 hours to prevent infection in 2 groups. From the second day after surgery, rehabilitation exercise was performed. All patients were taking X-ray examine before out of hospital. We suggest using brace for 1 month and avoid heavy physical activity within 3 months. This study was approved by the Human Ethics Committee of People’s Hospital of Xinjiang Uygur Autonomous Region. All patients provided written informed consent for endoscopic procedure.

### 2.1. Surgical technique

**MIS-TLIF:** After general anesthesia, the patient was placed prone position on the operating table. C-arm guidance was used to determine the disc space and mark the lateral pedicle line in the fluoroscopic anterior posterior view, and the lateral view was checked for tubular t.retractor system insertion (Medtronic and some Sanyou Ltd. equipment). Bilateral, paraspinal skin incisions were made approximately 2 cm from the midline and under the skin taking the Wlitse approach. Placed the Clark socket on the operating table rail and used the sterile fixed arm to secure the working channel in the incision with the help of the roving nurse. The illuminating device is installed in the working channel, and the 2-way opening makes the working channel easy to operate, and the extended device is used to complete the intervertebral space exposure, the nucleus pulposus removed and insert interbody fusion cage. Ultrasonic osteotome (Mazor) made total facetectomy, and the same side was fully decompressed and the contralateral side decompressed. A standard discectomy and preparation of disc was performed to allow insertion of the cage. Under fluoroscopic guidance, pedicle screws were then inserted and compression of the screws carried out as described previously. The incision can be sutured, no drainage tube is placed for it is just a single segment.

**CLIF:** The patient was placed in the right lateral position after general anesthesia, C-arm guidance was used to determine the disc space, and the target intervertebral space was marked. A 4 cm incision was made at the intersection of the intervertebral space line and the anterior tibiofibular line at the surgical segment. The incision direction was parallel to the extra-abdominal oblique muscle fibers, and perpendicularly intersected the anterior superior iliac spine and the umbilicus. The extra-abdominal oblique muscle, the intra-abdominal oblique muscle and the transverse abdominis muscle were bluntly separated along the fascia fibers into the retroperitoneal space, and the peritoneal, psoas muscle and large blood vessel sheath were bluntly separated by the finger along the posterior abdominal wall through the retroperitoneal space. Pull the psoas muscle back to explore the disc. At this time, insert the C-ring working tube (Shanghai Sanyou Company) along the direction of the finger to expand the layer by layer, expose the intervertebral disc and part of the vertebral body. Fluoroscopy guidance determine that the working tube is located at the target segment. The fiber loop was cut laterally, and the nucleus pulposus tissue and the upper and lower endplate tissues of the intervertebral space were removed with a curette, expose the subchondral bone clearly. Try the test Cage to find the right size, and the allogeneic bone were fully filled into the appropriate size of the Cage. Using the orthogonal principle to insert Cage perpendicular to the intervertebral space, use fluoroscopy checks the Cage position, make sure it is placed in the middle of the intervertebral space, and the intervertebral space and intervertebral foramen height increased. If necessary, it can be fixed laterally through the same working tube. Exit the working cannula, suture layer by layer, and use cosmetic suture. The incision can be sutured because the drainage tube is not placed in a single segment.

### 2.2. Special instrument

C-Ring pull hook system is the special instrument in lateral CLIF approach lumbar fusion of the cat’s eye, this system has unique self-stabilization and 1-way instantaneous stability function in design. It can flexibly open the muscles and avoid the damage of the psoas muscle and nerve plexus to the greatest extent. In different requirements, any pull tab can be adjusted at any time, a larger operating space, a clearer view of the operation. It was fixed on the vertebral body, avoiding the positioning change caused by the displacement of the patient during operation. The size of the 2, A set of tools can achieve CLIF surgery, and the system have 2 different types of diameter C-ring. A variety of length and diameter pull hook pieces, to meet different surgical styles and patient physical requirements, light-transparent material pull hook piece, better intervertebral condition during operation, and other bed frame interface to meet rigid fixation requirements

### 2.3. Keystone system

Keystone known as wedge stone side cage system (Fig. 2), which is designed from clinical, precise design, conforms to the forward tilt angle of Asian anatomy, it is also have accurately restores lumbar lordosis, unique length to width ratio, minimally invasive and safe, 3-dimensional anatomical surface, easier to implant, all these feature is to avoid the surrounding soft tissue and nerve’s injury and get good fusion. It has surface inverted tooth structure, effectively prevent the cage from exiting, original crosspiece design in the bone filling area, higher strength, larger bone grafting space, higher postoperative fusion rate.

**Figure 1.**
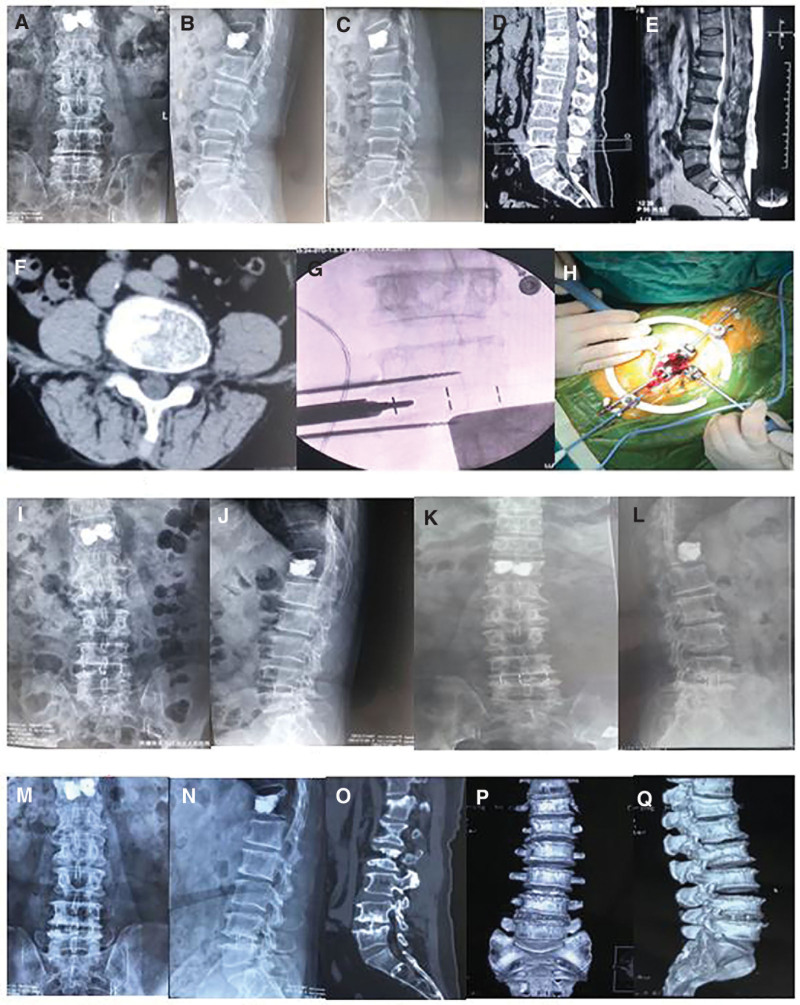
The patient was a 47 year old male, with low back pain and limited activity, without neurological symptoms of both lower limbs. His preoperative imaging examination (A–F), and He chosed CLIF operation (G, H). He underwent X-ray examination in 3 (I, J), 6 (K, L)and 12 (M–O)months after operation, and CT 3-dimensional reconstruction in 6 months after operation (P, Q). CLIF = Crenel lateral interbody fusion.

**Figure 2.**
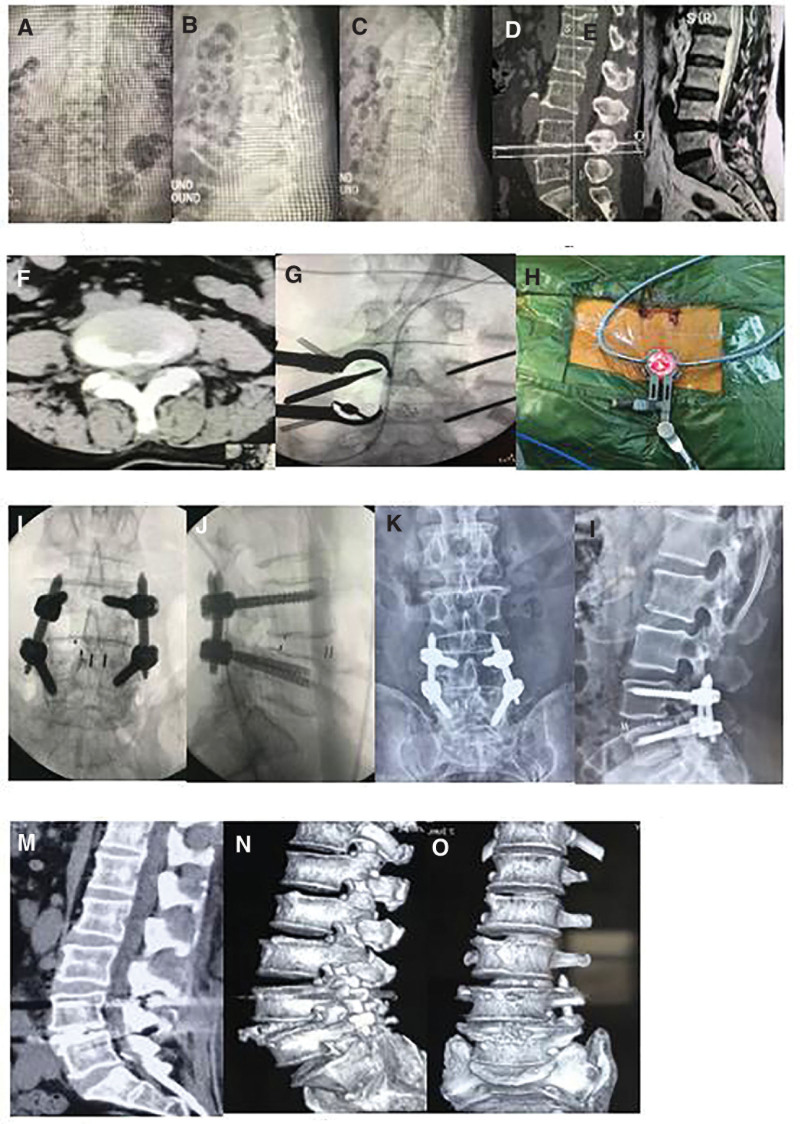
The patient was a 57 year old female, had recurrent low back pain with radiation pain of the right lower limb for 1 year and aggravated for 2 months. Her preoperative imaging examination (A–F), and She chosed MIS-TLIF operation (G, H). She underwent X-ray examination after operation (I, J) and reviewed in 3 (K, L) and 6 (M) months after operation, and CT 3-dimensional reconstruction in 6 months after operation (N, O). MIS-TLIF = minimally invasive transforaminal lumbar interbody fusion.

### 2.4. Outcome assessment

We observed operation time, intraoperative blood loss, postoperative drainage, bedtime and hospital stay, and postoperative complications for every patient. Low-back pain and leg pain scores were performed using visual analogue scores (VAS) 1 day before surgery, 7 days after surgery, and 3, 6 and 12 months after surgery, and the dysfunction index Oswestry disability index (ODI) was used, ODI score to assess treatment outcomes. X-ray films were evaluated for lumbar fusion according to the Bridwell^[3]^ method. Before the discharge from hospital and 3, 6 and 12 months after the operation, fusion was observed by X-ray, to find is there the presence or absence of cage subsidence, displacement and internal fixation failed happened.

### 2.5. Statistical analysis

Statistical analysis was carried out using the SPSS19.0 program for Windows V12.0 (SPSS Inc, Chicago, IL). Data were presented as the mean ± standard deviation. A probability value of less than 0.05 was considered significant. Independent 2 sample *t* test was used for the comparison of continuous variables between 2 groups. Paired *t* test was used to compare pre- and postoperative variables.

## 3. Results

All patients were followed up for 12 to 14 months, with an average of 12 months (Figs. 1 and 2). There were no significant differences in age, gender composition and segment between the 2 groups (*P* > .05).

### 3.1. Perioperative index

The operation time and intraoperative X-ray transmission time in the CLIF group were longer than those in the MIS group (*P* < .05). The intraoperative blood loss, postoperative drainage, bed rest, and hospitalization time were significantly lower in the CLIF group than in the MIS group (*P* < .01, Table 1). There was a case have numbness of the left lower extremity occurred 2 months after surgery in the CLIF group and at last had revision surgery.

**Table 1:** Comparison of perioperative parameters of the 2 groups of patients.

Group	Operation time (min)	Intraoperative blood loss (mL)	Intraoperative perspective (times)	Postoperative bed time (d)	Postoperative hospital stay (d)	Severe complications (n)
MIS, mean ± SD	132 ± 51	124 ± 53	10 ± 1.9	2.9 ± 0.8	6.5 ± 1.4	0
CLIF, mean ± SD	113 ± 28	73 ± 34	3 ± 0.7	1.8 ± 0.7	5.8 ± 0.8	1
t/X2	0.845	0.308	4.83	0.652	0.473	1.018
*P*	<.05	<.05	<.05	<.05	<.05	>.05

### 3.2. VAS and ODI scores

Low-back pain and leg pain VAS score taken in the CLIF group 7days and 3 months post operation, which were better than those in the MIS group. There was no significant difference between at the other time points in 2 group (*P* > .05). There was no significant difference in the ODI scores between the 2 groups at all time points (*P* > .05, Table 2).

**Table 2:** Comparison of VAS and ODI scoring results before and after operation.

Group	Low back pain VAS scores					leg pain VAS score					ODI score				
	Pre-operation	Postoperation	1 mo after operation	3 mo after operation	6 mo after operation	Pre-operation	7 d after operation	1 mo after operation	3 mo after operation	6 mo after operation	Pre-operation	Postoperation	1 mo after operation	3 mo after operation	6 mo after operation
MIS	5.6 ± 2.1	3.2 ± 0.8	3.0 ± 1.0	3.1 ± 1.0	1.7 ± 0.8	6.1 ± 1.8	3.8 ± 0.9	2.9 ± 1.0	2.7 ± 1.2	1.7 ± 0.9	50.13 ± 6.86	26.23 ± 5.65	18.55 ± 3.78	13.12 ± 3.17	10.53 ± 2.15
CLIF	5.1 ± 2.2	2.1 ± 0.8	2.0 ± 1.1	2.0 ± 1.1	1.9 ± 0.9	5.9 ± 1.9	3.2 ± 0.8	2.4 ± 0.9	2.3 ± 1.1	1.6 ± 0.7	49.17 ± 6.09	26.45 ± 3.17	17.26 ± 4.18	12.36 ± 2.46	9.86 ± 2.69
*P*	>.05	<.05	<.05	>.05	>.05	>.05	>.05	>.05	>.05	>.05	>.05	>.05	>.05	>.05	>.05

ODI = Oswestry disability index, VAS = visual analogue scores.

## 4. Imaging evaluation

According to Bridwell interbody fusion evaluation criteria, We compared twelve months after surgery. In MIS group, 19 cases at the grade I got fusion (72.7%), 9 cases at the grade II (29.3%) shows no cage displacement, losing and internal fixation failure from X-ray. In the CLIF group: 15 cases (66.7%) were at grade I, 12 cases (33.3%) were at grade II, and 1 case of numbness of the left lower limb after operation. There was case of cage losing and internal fixation failure happened. There was no significant difference in lumbar fusion rate between the 2 groups (*P* > .05).

### 4.1. Complication

There was 1 case of Cage sinking in the second month post-operation in CLIF group. At the end the case receive revision of TLIF operation and internal fixation. There were 2 cases (12.5%) had nerve injury and 1 case (6.25%) had transient psoas muscle weakness. There was no complication of wound infection, no breakage or failure of any screw or rod.

## 5. Discussion

Lumbar degenerative disease can be effectively treated by decompression or spinal fusion,^[4]^ in recent years lots of operation technique has been invented, including TLIF, MIS-TLIF and CLIF.^[4–9]^ Since Harms and Rolinger introduced a new technique to insert the interbody cage via the transforaminal route in 1982, TLIF has been introduced with the advancement of MIS.^[10]^ Although conventional MIS-TLIF has been proven to be a safe and effective surgical treatment among several fusion procedures available, few literature data are reported about clinical and radiological variables predictive to clinical results of CLIF. The traditional transforaminal interbody fusion uses a posterior median incision. The muscle attached to the spinous process is removed and pulled to the outside for pedicle screw placement, lamina decompression, fusion.^[11]^ Although this method can provide a good surgical vision, but it increases the muscle and soft tissue injury, more blood loss during operation, have more postoperative drainage, longer bed rest, slower postoperative recovery, etc.

MIS-TILF uses a latera-median incision to complete the intervertebral decompression and the placement of the cage with the aid of the working channel, reducing the stripping and pulling of muscle and soft tissue. Nowadays more studies have confirmed that the amount of bleeding and postoperative drainage during MIS-TLIF surgery is significantly less than open surgery, and the time to postoperatively and the length of hospital stay are less than that of open surgery.^[6–9]^

The anterior lateral CLIF approach, the lateral lumbar fusion of the cat’s eye, also through the psoas muscle intermuscular approach, with the newly designed C-Ring pull hook system for explore anterior and lateral side of lumbar disc and do the intervertebral fusion. C-Ring is used to expose the intervertebral disc and intervertebral space. It is not necessary to maintain excessive traction of the psoas muscle, and no neurological monitoring is needed to effectively prevent the occurrence of neurological complications. Skin incision is only about 4cm for single or 2 segment, C-Ring hook provides clear vision and enough operating space. As a lateral approach, to compared with the MIS-TLIF, CLIF also achieves the protective effect on the psoas muscle and the psoas muscle plexus. CLIF can directly do the decompression and end plate treatment, which is safer and more efficient. CLIF is a new way to treat lumbar degeneration, to avoid damage to the spinal nerve roots and reproductive femoral nerve.^[12]^ It is also a new way to deal with the degeneration of adjacent segments after lumbar internal fixation. The characterize of CLIF in surgery is to see the nerve directly, does not require intraoperative myoelectric monitoring, and through the use of a large Keystone system, can increase the intervertebral space, play an indirect decompression role.

Lumbar interbody fusion was fixed with lumbar fusion as the outcome. Studies have shown that the fusion rate of traditional MIS-TILF ranging from 91.8% to 99.0%.^[13]^ In this study, 12 months after surgery, according to Bridwell interbody fusion evaluation criteria at 12 months post-operation, MIS-TILF group: 19 cases of grade I fusion (72.7%), 9 cases of grade II (29.3%) without cage displacement, sinking, internal fixation Failure case. The CLIF group: 15 cases (66.7%) in grade I and 12 patients (33.3%) in grade II, compared with no significant difference.

In our study, the patient’s VAS score was restored from preoperative (5.1 ± 2.2) points to 12 months after surgery (1.9 ± 0.9) points; lumbar ODI score was restored from preoperative (49.17 ± 6.09) points to postoperative 12 months (9.86 ± 2.69) points were significantly improved compared with preoperative, there is no significant difference in the efficacy of CLIF and MIS-TLIF in the treatment of lumbar degenerative diseases, and satisfactory results can be obtained in both 2 groups. The results were similar to those reported in the literature.^[14]^ During the operation allogeneic bone grafting was used for every patient in CLIF group. Due to the choice of allogeneic bone, there is still a difference in long-term clinical result from autologous bone grafting in MIS-TLIF group. Regrettably, the long-term effect remains to be followed up. In this study, the operative time was 132 minutes in the MIS-TLIF group and 124 mL of blood loss; in the CLIF group, the blood loss was 73 mL, and operative time was 113 minutes. Some paper reported that the blood loss of MIS-TLIF was 124.4 mL, and the operation time was 115.8 minutes.^[15]^ In this study, the amount of blood loss in CLIF group was less than that of MIS-TLIF, and the operation time was shorter CLIF group, CLIF operation time was 113 ± 28 minutes, Intraoperative blood loss was 73 ± 34 mL.

Oliveria et al^[16]^ reported RCT of the indirect decompression effects of lateral approach intervertebral fusion. In his study a group of patients with degenerative disease and spinal stenosis (n = 21) only received a lateral approach for interbody fusion. Postoperative imaging evaluation showed that intervertebral height increases of 13.5%, intervertebral foramen area increased by 24.7%, and central spinal canal diameter increased by 33.1%. These results indicate that intervertebral distraction achieved by lateral approach interbody fusion is an effective mechanism for achieving decompression of degenerative spinal disease with stenotic symptoms. The biggest shortcoming of this study was the short follow-up period, with only 1 month of postoperative imaging. Sembrano et al^[17]^ found that the height of the intervertebral disc after TLIF increased (2.8 ± 3.7) mm. In this study the height of the leading and trailing edge of the intervertebral space increased (3.5 ± 2.4) mm and (3.3 ± 1.9) mm, respectively, it means CLIF superior to MIS-TLIF in increasing the height of intervertebral space, which may be related to the higher Cage height used in CLIF group than TLIF.

Diaz et al^[18]^ reported a group of 39 patients undergoing lateral approach interbody fusion (mean age 68 years), 4 of whom also underwent posterior internal fixation. Lateral placement of Cage can provide greater bone graft area than posterior fusion, but there is still the possibility of Cage sinking and shifting before thorough bone fusion. It happened also has positively correlated with Cage width, and the rate of sinking with width of 22 mm Cage was 1.9%, the rate of sinking with 18 mm Cage width was as high as 14.1%.^[19]^ In this study, there was a case of Cage sinking in the second month post-operation in CLIF group. At the end the case receive revision of TLIF operation and internal fixation. Considering the cause of failure may be related to the patient’s excessive treatment of the endplate during the operation, and osteoporosis is also very important reasons.

Nerve injury is 1 of the common surgical complications of lumbar interbody fusion. Different from traditional surgery, it is easily damage spinal canal and nerve root canal during decompression. MIS-TLIF surgery need to pull the psoas muscle and easily damage theplexus, CLIF surgery damage the reproductive femoral nerve possibility, and cause leg pain and paresthesia in the lower extremities, and numbness, weakness. Mehren et al^[20]^ reported 3 cases of neurological injury after MIS-TLIF, accounting for 0.37%; In this study, there were 2 cases (12.5%) had nerve injury and 1 case (6.25%) had transient psoas muscle weakness. The symptoms disappeared after 3 months of follow-up. No damage to the femoral nerve was seen in the study.

## 6. Conclusion

In summary, the indications for CLIF are similar to MIS-TLIF, the post-operation outcome and fusion rate are comparable in 12 months after surgery. Surgery for revision surgery and adjacent spondylosis is superior to MIS-TLIF surgery, providing a new the revision method, the operation time is reduced, and the vertebral space and the height of the intervertebral foramen are better than MIS-TLIF. The complication rate is to be observed according to the long-term follow-up time. CLIF is an effective method for the treatment of lumbar degenerative diseases. It is a side approach, so it is impossible to operate in the gap of the lumber 5^th^ and sacrum1st, so indication is very important. To further understanding of differences in outcomes following CLIF and MIS-TLIF, prospective, long-term studies are necessary. However, there is a limitation that the sample size might be not large enough to find the more existing evidence that CLIF was superior to MIS-TLIF. Till now, the significance of CLIF remains known based on this study, but it limited data available from other study, due to it was a new way to treat lumbar degeneration.

## Author contributions

**Conceptualization:** Chenxi Li, Hao Wang.

**Data curation:** Shaxika·Nazierhan.

**Formal analysis:** Chenxi Li, Rui Guo, Linsong Lu, Kuo Xu.

**Investigation:** Shaxika·Nazierhan, Rui Guo, Dilimulati·Aikeremu.

**Methodology:** Rui Guo.

**Project administration:** Linsong Lu, Dilimulati·Aikeremu.

**Resources:** Rui Guo.

**Software:** Chenxi Li, Dilimulati·Aikeremu.

**Supervision:** Dilimulati·Aikeremu, Kuo Xu.

**Validation:** Kuo Xu, Hao Wang.

**Writing – original draft:** Shaxika·Nazierhan.

**Writing – review & editing:** Chenxi Li, Hao Wang.
